# FUS/TLS Suppresses Enterovirus Replication and Promotes Antiviral Innate Immune Responses

**DOI:** 10.1128/JVI.00304-21

**Published:** 2021-05-24

**Authors:** Yuan Chao Xue, Chen Seng Ng, Yasir Mohamud, Gabriel Fung, Huitao Liu, Amirhossein Bahreyni, Jingchun Zhang, Honglin Luo

**Affiliations:** a Centre for Heart and Lung Innovation, St. Paul’s Hospital, University of British Columbia, Vancouver, British Columbia, Canada; b Department of Pathology and Laboratory Medicine, University of British Columbia, Vancouver, British Columbia, Canada; c Department of Experimental Medicine, University of British Columbia, Vancouver, British Columbia, Canada; Instituto de Biotecnologia/UNAM

**Keywords:** RNA binding proteins, enterovirus, host response

## Abstract

During viral infection, the dynamic virus-host relationship is constantly in play. Many cellular proteins, such as RNA-binding proteins (RBPs), have been shown to mediate antiviral responses during viral infection. Here, we report that the RBP FUS/TLS (fused in sarcoma/translocated in liposarcoma) acts as a host-restricting factor against infection with coxsackievirus B3 (CVB3). Mechanistically, we found that deletion of FUS leads to increased viral RNA transcription and enhanced internal ribosome entry site (IRES)-driven translation, with no apparent impact on viral RNA stability. We further demonstrated that FUS physically interacts with the viral genome, which may contribute to direct inhibition of viral RNA transcription/translation. Moreover, we identified a novel function for FUS in regulating host innate immune response. We show that in the absence of FUS, gene expression of type I interferons and proinflammatory cytokines elicited by viral or bacterial infection is significantly impaired. Emerging evidence suggests a role for stress granules (SGs) in antiviral innate immunity. We further reveal that knockout of FUS abolishes the ability to form SGs upon CVB3 infection or poly(I·C) treatment. Finally, we show that, to avoid FUS-mediated antiviral response and innate immunity, CVB3 infection results in cytoplasmic mislocalization and cleavage of FUS through the enzymatic activity of viral proteases. Together, our findings in this study identify FUS as a novel host antiviral factor which restricts CVB3 replication through direct inhibition of viral RNA transcription and protein translation and through regulation of host antiviral innate immunity.

**IMPORTANCE** Enteroviruses are common human pathogens, including those that cause myocarditis (coxsackievirus B3 [CVB3]), poliomyelitis (poliovirus), and hand, foot, and mouth disease (enterovirus 71). Understanding the virus-host interaction is crucial for developing means of treating and preventing diseases caused by these pathogens. In this study, we explored the interplay between the host RNA-binding protein FUS/TLS and CVB3 and found that FUS/TLS restricts CVB3 replication through direct inhibition of viral RNA transcription/translation and through regulation of cellular antiviral innate immunity. To impede the antiviral role of FUS, CVB3 targets FUS for mislocalization and cleavage. Findings from this study provide novel insights into interactions between CVB3 and FUS, which may lead to novel therapeutic interventions against enterovirus-induced diseases.

## INTRODUCTION

During viral infection in eukaryotic cells, the virus-host relationship is a dynamic tug-of-war in which each attempts to suppress the other. Typically, during infection with RNA viruses (such as enteroviruses), viral particles first enter the cells through endocytosis, escape the encapsulated membranes while releasing the viral RNA from its protected icosahedral capsid composed of 4 structural proteins (VP1 to VP4), and subsequently hijack cellular machineries for viral replication/expansion (1). While the viral infection process is unfolding, cellular pathogen recognition receptors (PRRs) recognize specific foreign moieties such as viral RNA, which initiate the induction of innate immune responses, as reflected by increased production of interferons (IFNs) and interferon-stimulated genes (ISGs) (2).

RNA-binding proteins (RBPs), among many other cellular proteins, are widely established as playing either antiviral or proviral roles. For example, TAR DNA-binding protein 43 (TDP-43) and ARE/poly(U)-binding/degradation factor 1 (AUF1/hnRNP D) have both been shown to play an antiviral role against various enteroviruses, and to overcome this effect, viruses have adopted different strategies to disrupt the function of these RBPs (3–8). Another functionally and structurally relevant RBP is FUS/TLS (fused in sarcoma/translocated in liposarcoma); however, the role of FUS in viral infection and the interplay between FUS and enteroviruses have not been explored (9). FUS has been studied widely, first in the context of cancer as an oncoprotein in myxoid liposarcomas and later as one of the causative genes in the development of amyotrophic lateral sclerosis (ALS), or Lou Gehrig’s disease (10–12). To date, FUS has been linked to many important cellular functions, such as transcription, RNA splicing, translation, and DNA repair (10–12).

In this study, we examined the role of FUS in enteroviral infection using coxsackievirus B3 (CVB3) as a model by investigating both sides of this virus-host relationship. We found that CVB3 mislocalizes FUS from its nuclear location to the cytoplasm and then targets it for degradation through the action of virus-encoded proteases. Further investigation revealed that FUS acts as a host restriction factor inhibiting viral RNA transcription and cap-independent viral protein translation and that loss of FUS as a result of CVB3 infection causes enhanced viral replication. Finally, we also identified a novel function for FUS in regulating innate immune response and stress granule (SG) formation, which likely contributes, at least in part, to its antiviral activity.

## RESULTS

### CVB3 infection results in cytoplasmic mislocalization and cleavage of FUS.

**FIG 1:**
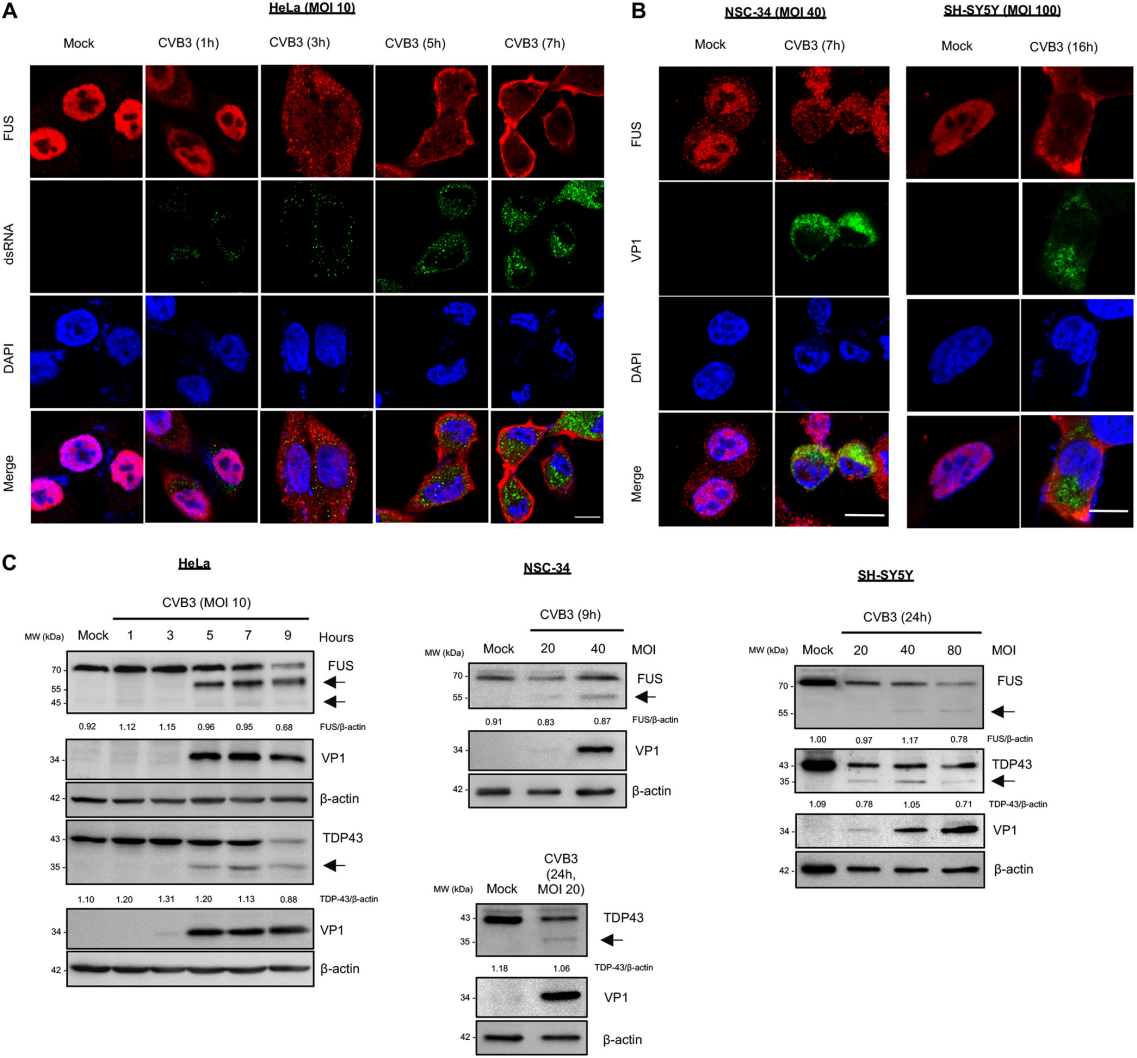
CVB3 infection results in cytoplasmic mislocalization and cleavage of FUS. (A and B) HeLa cells (A) and NSC-34 and SH-SY5Y cells (B) were infected with CVB3 at an MOI of 10, 40, or 100, respectively, for various times as indicated. Cells were then fixed and immunostained for FUS (red) and dsRNA (green). Nuclei were counterstained with DAPI (blue). Bar = 10 μm. (C) HeLa, NSC-34, and SH-SY5Y cells were infected with CVB3 at different MOIs for various times, as indicated. Western blotting was conducted for detection of FUS, VP1, and β-actin (protein loading control). TDP-43 was probed as a positive control for viral protease-mediated cleavage. Protein levels of full-length FUS and TDP-43 were quantitated by densitometric analysis using Fiji image analysis software and normalized to levels of β-actin and are presented as fold changes underneath the individual Western blots. Arrows denote the possible cleavage fragments. This cleavage experiment was repeated more than three times.

Our and other labs showed previously that some RBPs, such as TDP-43 and AUF1, exert antiviral effects on enteroviruses, and these findings prompted us to further investigate the relationship of another functionally and structurally related RBP, FUS, with enteroviral infection (3–8). We found that after infection with CVB3, a neurotrophic virus, FUS was mislocalized from its normal nuclear location to the cytoplasm starting at 3 h postinfection in HeLa cells (human cervical cancer cells), NSC-34 cells (mouse motor neuron-like cells), and SH-SY5Y cells (human neuroblastoma cells) (Fig. 1A and B). We further conducted Western blot analysis to examine protein expression of FUS upon CVB3 infection. Figure 1C shows that protein levels of full-length FUS were gradually reduced after CVB3 infection, accompanied by the generation of two possible cleavage fragments (55 kDa and 45 kDa), with the 55-kDa band being more dominant and consistently seen in all three cell models. It was noted that the appearance of these low-molecular-weight, FUS antibody-reactive bands occurred concurrently with the detection of viral protein VP1, suggesting an event dependent on viral replication. The cleavage of TDP-43 was examined and is shown here as a positive control (4).

### Expression of CVB3 proteases leads to mislocalization and cleavage of FUS.

**FIG 2:**
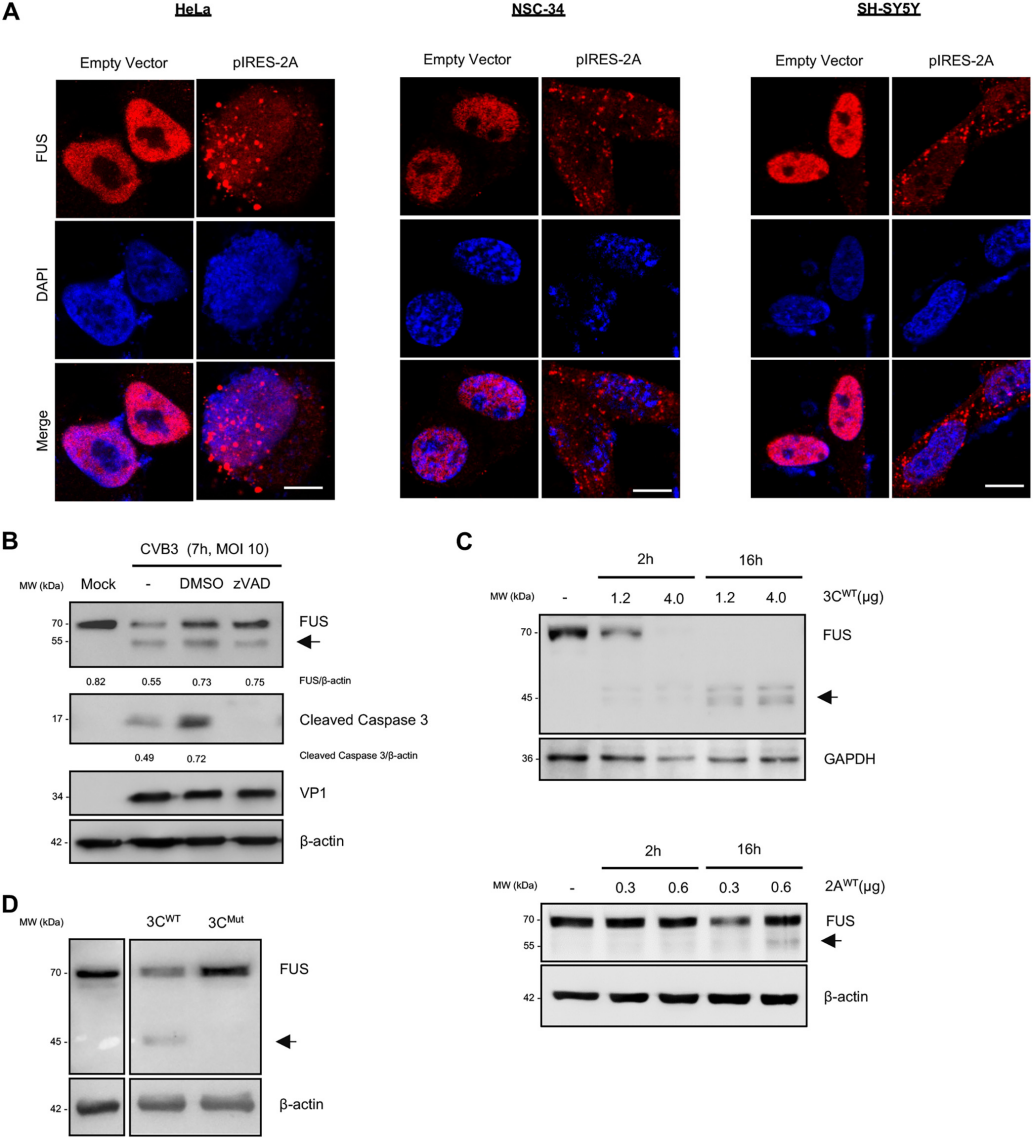
Expression of CVB3 proteases leads to mislocalization and cleavage of FUS. (A) HeLa, NSC-34, and SH-SY5Y cells were transiently transfected with empty vector or pIRES-2A for 24 h. Immunofluorescent staining was performed to examine the expression and distribution of FUS (red). Nuclei were counterstained with DAPI (blue). Bar = 10 μm. (B) HeLa cells were treated with 50 μM zVAD (a pan-caspase inhibitor) or dimethyl sulfoxide (DMSO) during the 7-h CVB3 infection (MOI, 10). Western blotting was conducted to assess the expression of FUS, cleaved caspase 3, VP1, and β-actin, followed by densitometric analysis as described in the legend to Fig. 1. (C) *In vitro* (2A and 3C) viral protease cleavage assays were carried out by incubating HeLa cell lysates (30 μg) with the indicated amounts of purified recombinant CVB3 2A or 3C at 37°C for 2 or 16 h as indicated. Western blotting was performed to examine protein expression of FUS and GAPDH/β-actin (loading control). (D) HeLa cells were transiently transfected with WT or catalytically inactive mutant 3C plasmids (1 μg) for 24 h, followed by Western blot analysis of FUS and β-actin.

Next, we questioned whether viral and/or cellular proteases mediate mislocalization and cleavage of FUS. CVB3 encodes two viral proteases (3C and 2A) that are responsible for cleaving both the viral and host proteins during viral replication (13). Of the two, 2A has been shown previously to play a major role in disrupting the nuclear pore complex through cleavage of key proteins such as nucleoporins 62 (NUP62), 98, and 152 (14, 15), leading to the leakage of nuclear proteins into the cytoplasm. To determine whether mislocalization of FUS is mediated through the action of viral protease 2A, we transiently transfected an IRES-driven 2A (pIRES-2A) plasmid in HeLa, NSC-34, and SH-SY5Y cells. We demonstrated that FUS was localized to the cytoplasm in cells expressing pIRES-2A while remaining in the nucleus in control cells (Fig. 2A). This finding reflects the observation made in CVB3-infected cells, indicating that that 2A is responsible for CVB3-mediated FUS mislocalization.

During enteroviral infection, cellular caspases are activated, leading to the cleavage of multiple cellular proteins as a way to enhance viral particle release (16). Therefore, it was important to first distinguish if the observed FUS cleavage (Fig. 1C) is due to activated caspases or viral proteases. HeLa cells were infected with CVB3 in the presence of zVAD (a pan-caspase inhibitor) for 7 h. We observed that caspase inhibition, as evidenced by the absence of cleaved caspase 3, after addition of zVAD did not prevent the cleavage of FUS, suggesting that these fragments are likely mediated by viral proteases (Fig. 2B). We then performed *in vitro* and *in vivo* cleavage assays by incubating HeLa cells lysates with recombinant CVB3 3C and 2A (Fig. 2C) and by transiently transfecting viral protease plasmids in HeLa cells (Fig. 2D), respectively. We found that both 3C and 2A proteases were capable of cleaving FUS, with 3C being responsible for the production of the 45-kDa fragment (Fig. 2C, top, and Fig. 2D) and 2A being responsible for the generation of the more dominant 55-kDa fragment (Fig. 2C, bottom).

### Knockdown or knockout of FUS promotes viral infection.

**FIG 3:**
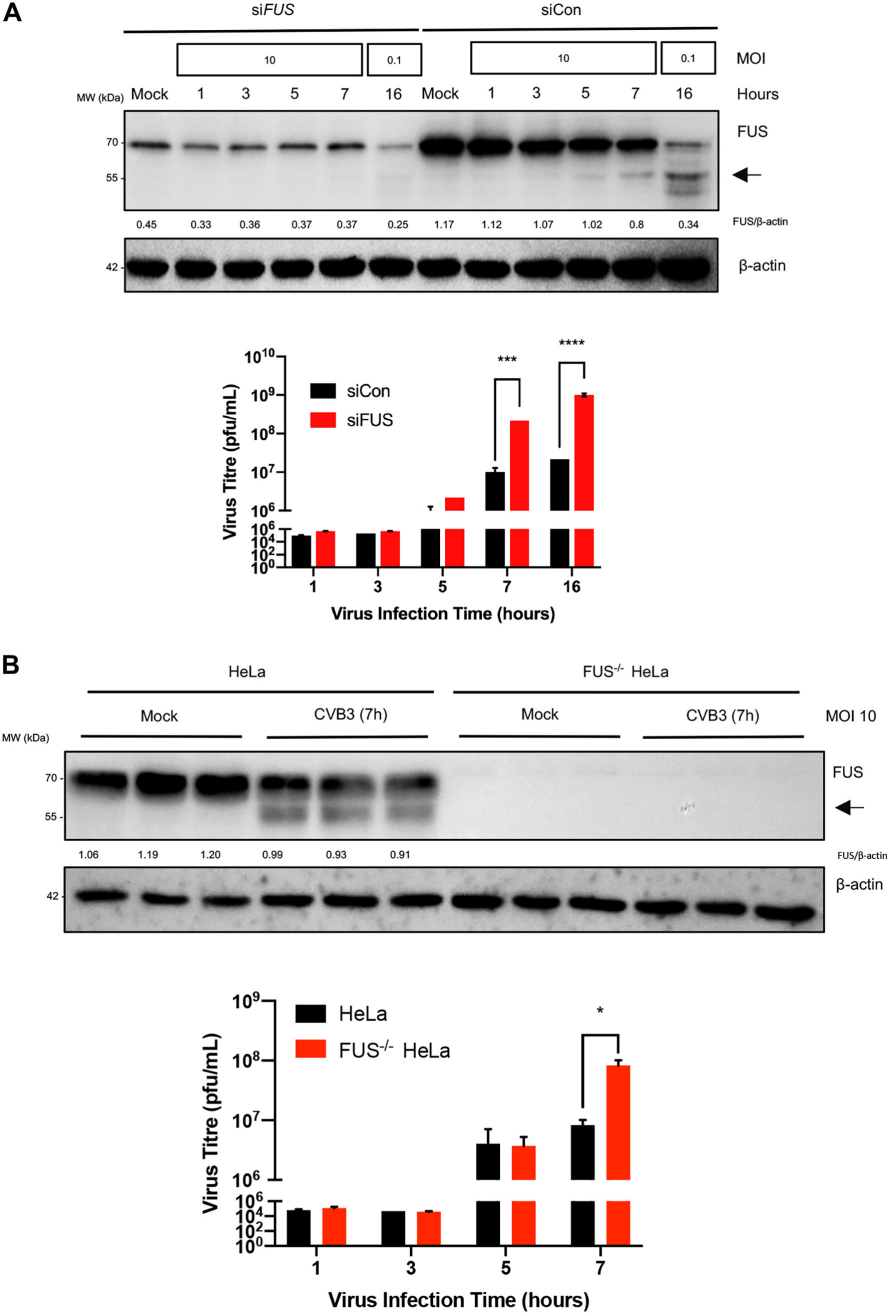
Knockdown or knockout of FUS promotes viral infection. (A) HeLa cells were transfected with scramble control siRNA (siCon) or FUS-specific siRNA (siFUS) for 48 h, followed by infection with CVB3 at an MOI of 10 for 1, 3, 5, or 7 h or at an MOI of 0.1 for 16 h. FUS cleavage and FUS knockdown efficiency were confirmed by Western blotting. Virus titers in the supernatants were measured using a virus titration assay (*n* = 3). (B) Control and FUS knockout (FUS^−/−^) HeLa cells established by CRISPR-Cas9 gene editing were infected with CVB3 as described above. FUS knockout and cleavage were verified by Western blotting. Supernatants were collected, and virus titers were measured by virus titration assay (*n* = 3). Protein expression was quantified as described for Fig. 1. Statistical analysis was performed by unpaired Student's *t* test. *, *P* < 0.05; ***, *P* < 0.0005; ****, *P* < 0.0001.

We next sought to determine the functional consequence of loss of FUS during viral infection. We knocked down FUS in HeLa cells using small interfering RNA (siRNA) and utilized FUS knockout (FUS^−/−^) HeLa cells established through CRISPR-Cas9 gene editing for this study. As shown in Fig. 3, virus titration assay revealed that viral titers were significantly higher (>10-fold increase) in cells with FUS knockdown (Fig. 3A) or knockout (Fig. 3B) than in control cells that express normal levels of FUS. This result suggests that FUS is a cellular antiviral factor during CVB3 infection.

### Knockout of FUS causes increased viral RNA transcription and enhanced IRES-dependent translation.

**FIG 4:**
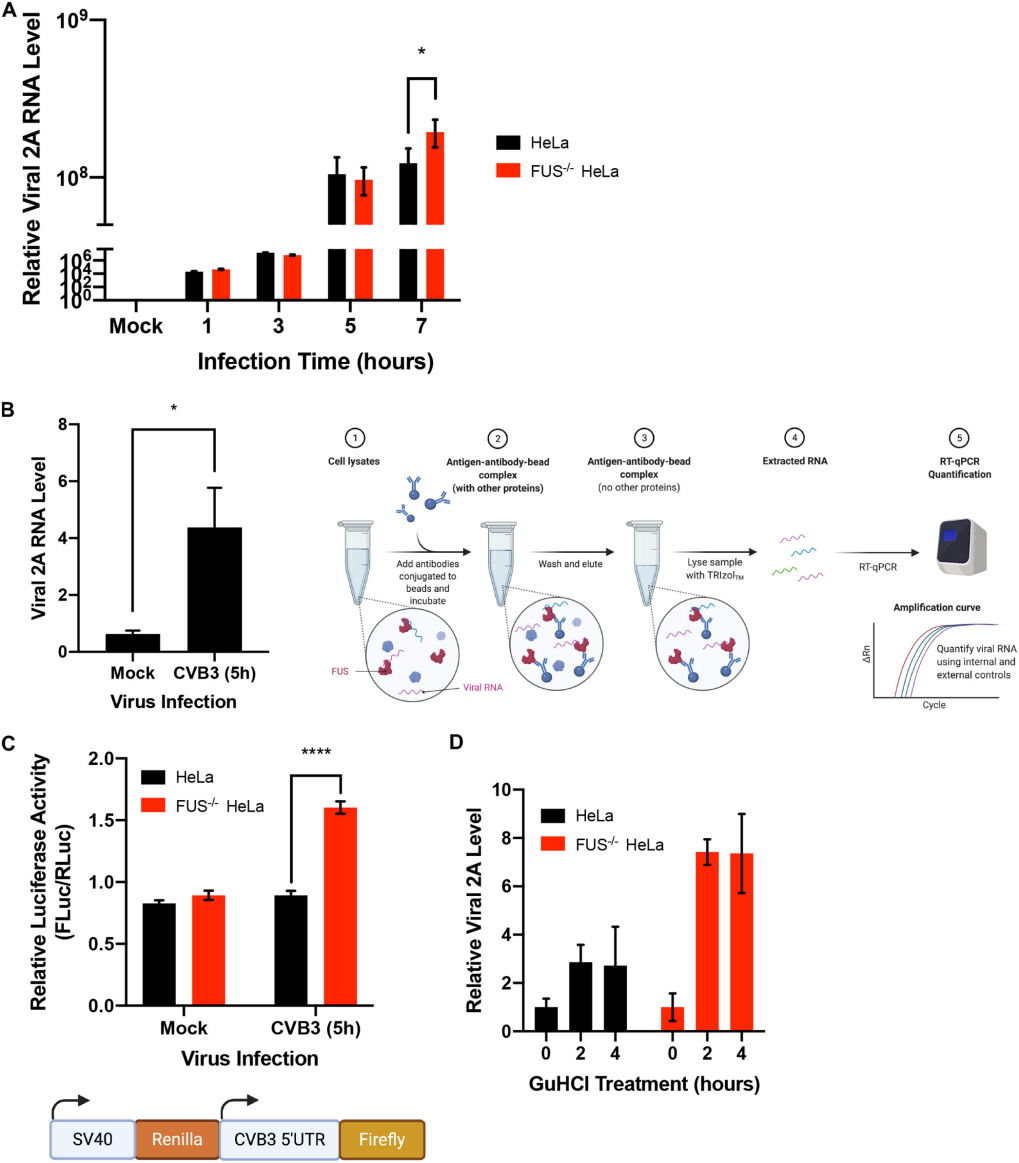
Knockout of FUS causes increased viral RNA transcription and enhanced IRES-dependent translation. (A) Control and FUS^−/−^ cells were infected with CVB3 (MOI 10) for 1, 3, 5, or 7 h, and the relative viral RNA levels were measured by qPCR using primer pairs targeting the viral 2A gene and then normalized to *GAPDH* RNA levels (*n* = 3). (B) HeLa cells were either mock or CVB3 infected (MOI, 10) for 5 h. FUS immunoprecipitation was then conducted, followed by qPCR measurement of CVB3 RNA levels on eluted RNA samples using primer pairs targeting the CVB3 2A gene region (schematic diagram of the procedure on the right; *n* = 3). (C) Control and FUS^−/−^ HeLa cells were transfected with the C49-CVB3-5′UTR dual luciferase reporter plasmid for 24 h, followed by CVB3 infection (MOI, 10) for 5 h. Lysates were collected, and the dual-luciferase reporter assay was conducted following the manufacturer’s instructions. The results are presented as the relative luciferase activity of firefly luciferase (CVB3-5′UTR; FLuc) over *Renilla* luciferase (SV40 internal control; RLuc) (*n* = 3). (D) Control and FUS^−/−^ HeLa cells were infected with CVB3 (MOI, 10) for 5 h and then treated with 2 mM GuHCl for an additional 2 or 4 h. qPCR was performed to measure relative viral RNA levels using primer pairs targeting CVB3 2A gene region and then normalized to *GAPDH* RNA levels (*n* = 3). Statistical analysis was performed by unpaired Student's *t* test. ****, *P* < 0.0001.

To understand the underlying antiviral mechanism of FUS, we first investigated the impacts of FUS deletion on viral RNA expression. As an RBP, FUS interacts with cellular RNAs and regulates their transcription, stability, and translation (9, 17, 18). We suspected that FUS affects viral replication by interfering with the viral RNA transcription. We found that, similar to the virus titer results shown in Fig. 3, viral RNA levels were significantly higher (∼2-fold increase) in FUS^−/−^ cells than control cells at 7 h postinfection, suggesting that FUS plays a role in suppressing the expression of viral RNA (Fig. 4A). Moreover, an RNA immunoprecipitation assay demonstrated that FUS was able to bind to viral RNA (Fig. 4B), suggesting a possible mechanism by which FUS inhibits viral growth through direct interaction with the viral RNAs.

Enteroviral RNA translation is governed by the activity of viral internal ribosome entry site (IRES) within the 5′ untranslated region (UTR) (19). To determine whether FUS also has a role in regulating IRES-mediated viral translation, we transiently transfected a dual-luciferase expression vector, C49-CVB3-5′UTR (20), into control and FUS^−/−^ HeLa cells for 24 h, followed by mock or CVB3 infection for 5 h. The C49-CVB3-5′UTR reporter plasmid contains a simian virus 40 (SV40)-driven *Renilla* luciferase (RLuc) coding sequence that acts as an internal cap-dependent translation control and a CVB3-specific 5′ UTR-driven firefly luciferase (FLuc) coding sequence that measures cap-independent protein translation (Fig. 4C). We discovered that the luminescence ratio of FLuc to RLuc (CVB3 5′ UTR/SV40) was significantly higher (∼1.5-fold increase) in FUS^−/−^ than in control HeLa cells, suggesting that FUS can suppress CVB3 IRES-driven translation during CVB3 infection (Fig. 4C).

It was previously reported that FUS can also affect cellular RNA stability (18); therefore, we questioned whether the increased levels of viral RNA observed in FUS^−/−^ cells are a result of enhanced viral RNA stability. Experimentally, we infected FUS^−/−^ and control HeLa cells with CVB3 for 5 h, followed by guanidine hydrochloride (GuHCl; an inhibitor of picornaviral replication by blocking the initiation of viral RNA synthesis [21]) treatment for 2 and 4 h. Under these conditions, the inhibition of viral RNA expression by GuHCl allows the measurement of the viral RNA degradation rate. It is expected that if FUS affects CVB3 RNA stability in FUS^−/−^ HeLa cells, the 4-h treatment group would have a viral RNA level different from that of the 2-h treatment group. However, we did not observe a significant difference in the viral RNA levels in control and FUS^−/−^ HeLa cells after both 2- and 4-h treatments (Fig. 4D). Together, our results indicate that FUS plays an antiviral role through inhibiting viral RNA transcription and IRES-driven translation, without evident effects on viral RNA stability.

### Knockout of FUS results in decreased gene production of type I IFN and inflammatory cytokines in response to various stimulations.

**FIG 5:**
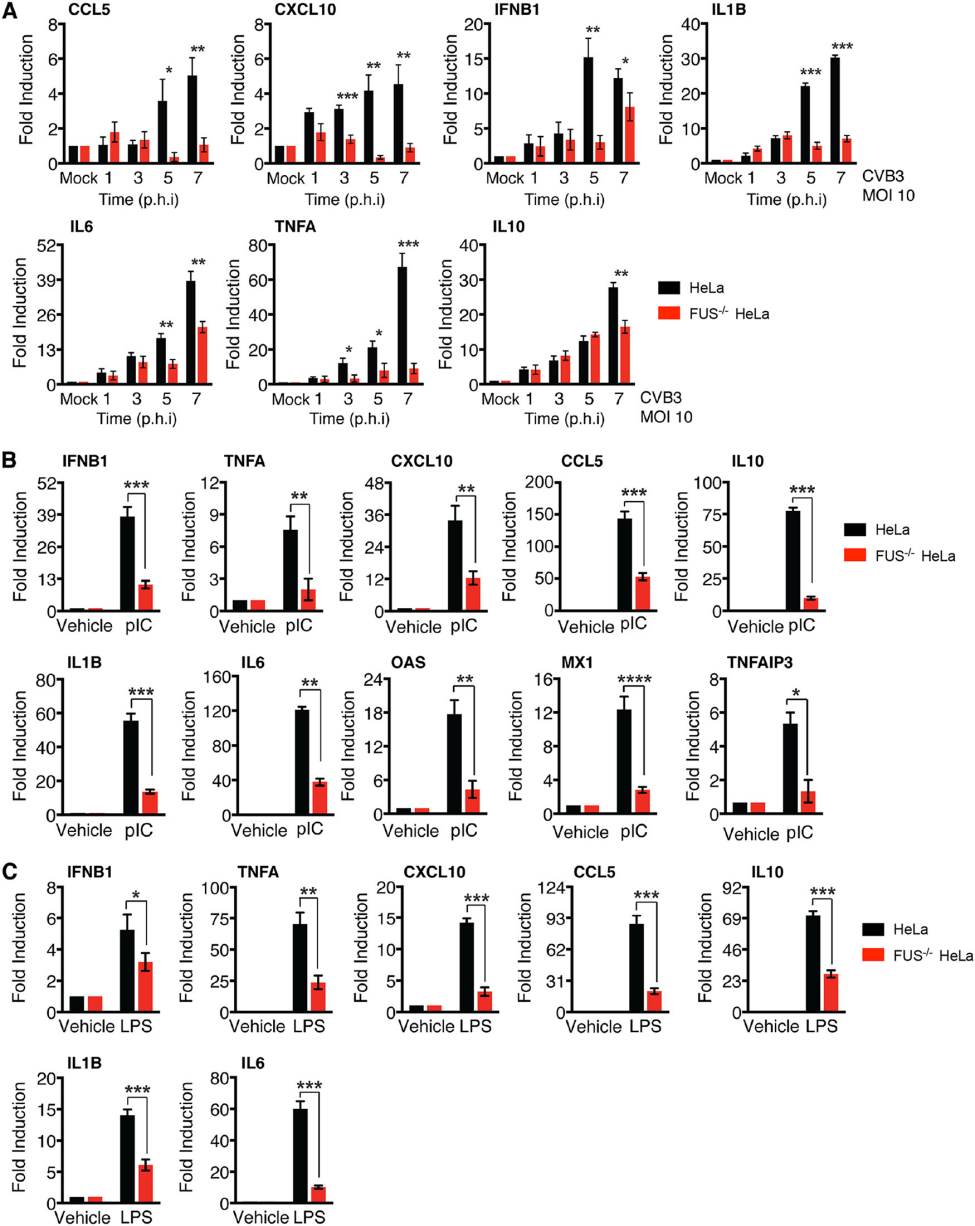
Knockout of FUS results in increased gene production of IFN-I and inflammatory cytokines in response to various stimulations. Control and FUS^−/−^ HeLa cells were either mock infected or infected with CVB3 (MOI 10) for 1, 3, 5, and 7 h (A) or treated with vehicle or poly(I·C) (1 μg/ml) for 6 h (B) or LPS (100 ng/ml) for 5 h (C). Gene expression of IFN-I and the indicated inflammatory cytokines was measured by RT-qPCR. The results were normalized to *GAPDH* RNA levels and then presented as fold changes relative to RNA levels in mock-infected or vehicle-treated groups, which were arbitrarily set to 1.0 (*n* = 3). Statistical analysis was performed by unpaired Student's *t* test. *, *P* < 0.05; **, *P* < 0.005; ***, *P* < 0.0005.

Type I interferons (IFN-I) play an essential role in the innate immune response against viral infections by targeting multiple steps of the viral life cycle, including viral RNA transcription and translation (22). We then decided to determine, in addition to its direct effects on viral gene expression and translation, whether FUS can also exert its antiviral function by regulating the innate immune response. We measured the RNA levels of IFN-I and various inflammatory cytokines, as indicated in Fig. 5, in FUS^−/−^ and control cells infected with CVB3 (Fig. 5A) and treated with poly(I·C), a chemical mimic of viral double-stranded RNA (dsRNA) (Fig. 5B) or lipopolysaccharide (LPS) from Escherichia coli O111:B4, the major membrane component of the Gram-negative bacteria (Fig. 5C). With all the stimulants tested, RNA levels of IFN-I and inflammatory cytokines were significantly lower in FUS^−/−^ cells than control cells, suggesting a novel role for FUS in controlling global innate immune responses.

### Knockout of FUS significantly decreases CVB3- and poly(I.C)-induced SG formations.

**FIG 6:**
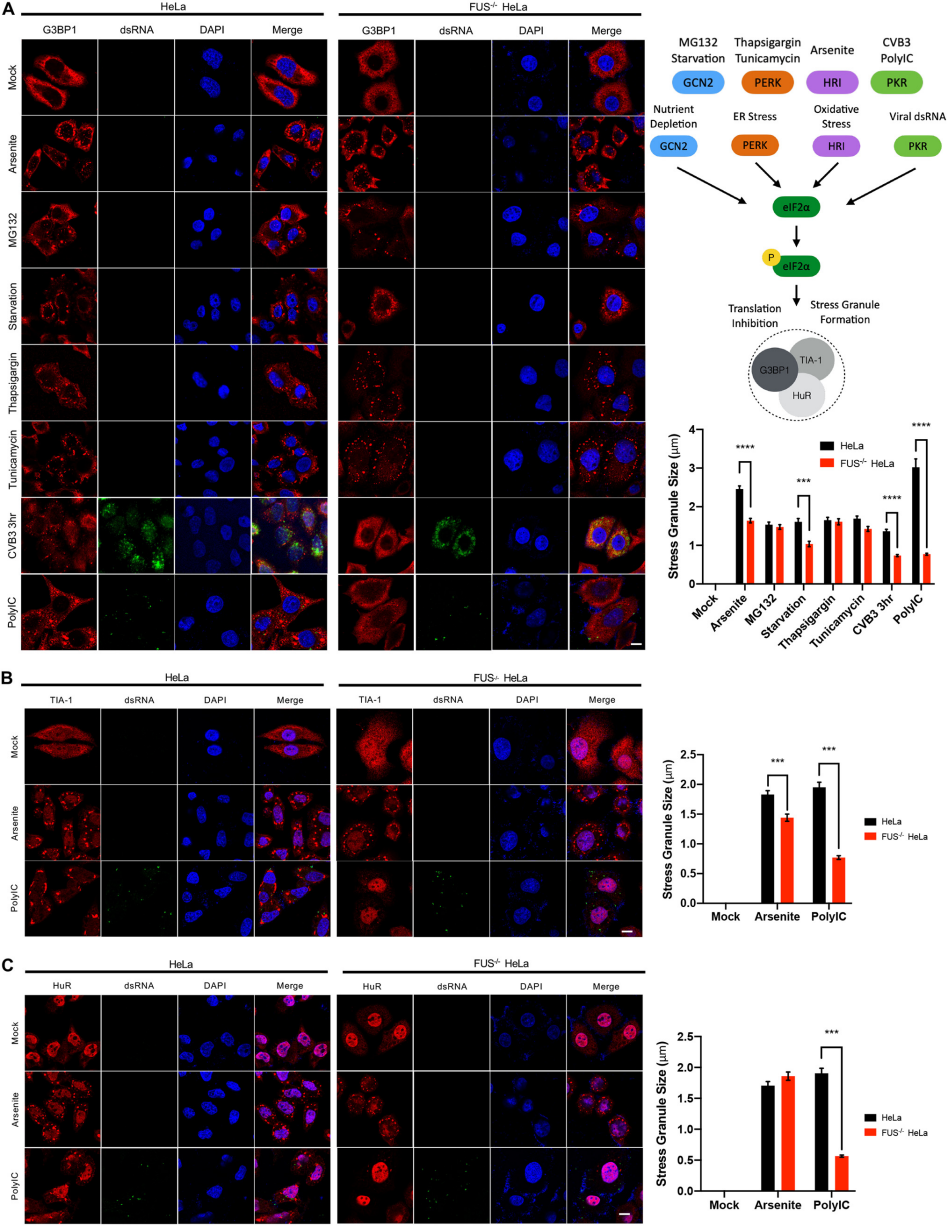
Knockout of FUS significantly decreases CVB3- and poly(I·C)-induced SG formation. (A) Control and FUS^−/−^ HeLa cells were treated with arsenite (0.5 mM) for 1 h, MG132 (10 μM) for 3 h, HBSS starvation medium for 6 h, thapsigargin (4 μM) for 1 h, tunicamycin (1 μg/ml) for 6 h, CVB3 (MOI, 10) for 3 h, or poly(I·C) (1 μg/ml) for 6 h. Subsequently, cells were fixed and immunostained for G3BP1, dsRNA, and DAPI. The size of SGs, marked by G3BP1 puncta, was quantified using a total of 100 SGs in 3 biological replicates, taking an average of 5 SGs/cell (*n* = 100). The schematic depiction of 4 common pathways and associated stimuli leading to the formation of SGs is shown on the upper right. (B and C) Control and FUS^−/−^ HeLa cells were treated with either arsenite (0.5 mM) for 1 h or poly(I·C) (1 μg/ml) for 6 h and then immunostained for TIA-1 and dsRNA (B) or HuR and dsRNA (C). The size of SGs, marked by TIA-1 or HuR puncta, was measured and quantified as described above. Statistical analysis was performed by unpaired Student's *t* test. ***, *P* < 0.0005; ****, *P* < 0.0001. Bar = 10 μm.

The observation of granule-like structures of FUS (Fig. 1A and 2A), the involvement of FUS in antiviral IFN-I response (Fig. 5), and the recognized role of SGs in antiviral innate immunity (23, 24) prompted us to test if FUS utilizes SGs as a platform for amplifying/exerting the immune responses against CVB3. Immunofluorescent staining was conducted on cells under various stress conditions known to trigger SG formation through different signaling pathways (Fig. 6A, top right) (25). We found that all of the stimulants, with the exception of CVB3 and poly(I·C), were capable of inducing SG formation, measured by G3BP1 puncta, in both control and FUS^−/−^ cells. In FUS^−/−^ cells, deletion of FUS almost abolished CVB3- and poly(I·C)-triggered SG formation compared to HeLa cells (Fig. 6A). This phenomenon was also observed in cells treated with poly(I·C) and stained for TIA-1 (Fig. 6B) and HuR (Fig. 6C), two other core components of the SGs. Collectively, our results indicate that FUS plays a role in CVB3/poly(I·C)-induced SG formation, which may contribute to its antiviral immune response.

### Reintroduction of FUS into FUS knockout cells rescues poly(I.C)-induced stress granule formation and IFN-β and TNF-α gene expression.

**FIG 7:**
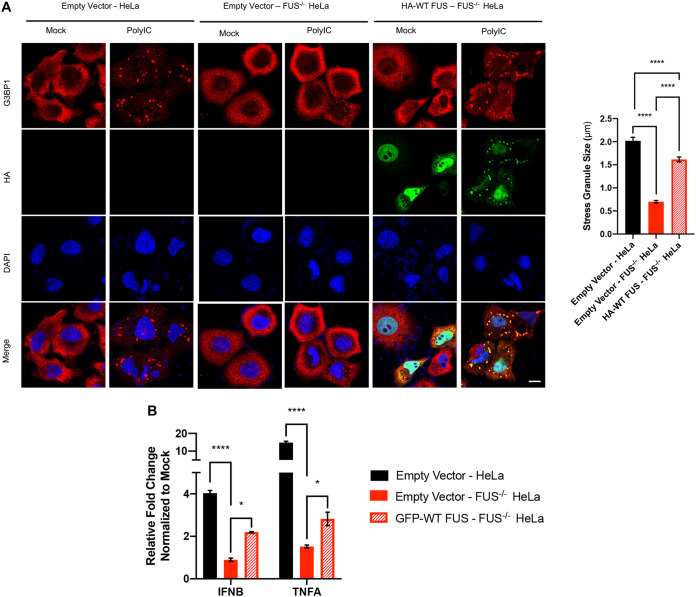
Reintroduction of FUS into FUS knockout cells rescues poly(I·C)-induced SG formation and expression of IFN-β and TNF-α genes. Control and FUS^−/−^ HeLa cells were transfected with empty vector or HA-tagged WT-FUS for 24 h, followed by mock or poly(I·C) (1 μg/ml for 6 h) treatment. (A) Cells were immunostained for G3BP1, HA, and DAPI. The size of SGs, marked by G3BP1 puncta, was measured and quantified as described for Fig. 6. Bar = 10 μm. (B) Expression of *IFNB* and *TNFA* in cells was measured by qPCR. Statistical analysis was performed by two-way analysis of variance (ANOVA) followed by Tukey’s multiple-comparison test. *, *P* < 0.05; ****, *P* < 0.0001.

We have so far demonstrated that in the absence of FUS, the antiviral innate immune response is damped, which is likely mediated through abolishment of SG formation. To verify this antiviral mechanism of FUS, we reintroduced FUS into FUS^−/−^ cells by transient transfection and found that expression of FUS rescued poly(I·C)-induced SG formation (Fig. 7A) and *IFNB* and *TNFA* expression (Fig. 7B), confirming a key role for FUS in SG formation and the innate immune response.

### Knockout of FUS inhibits autophagy.

**FIG 8:**
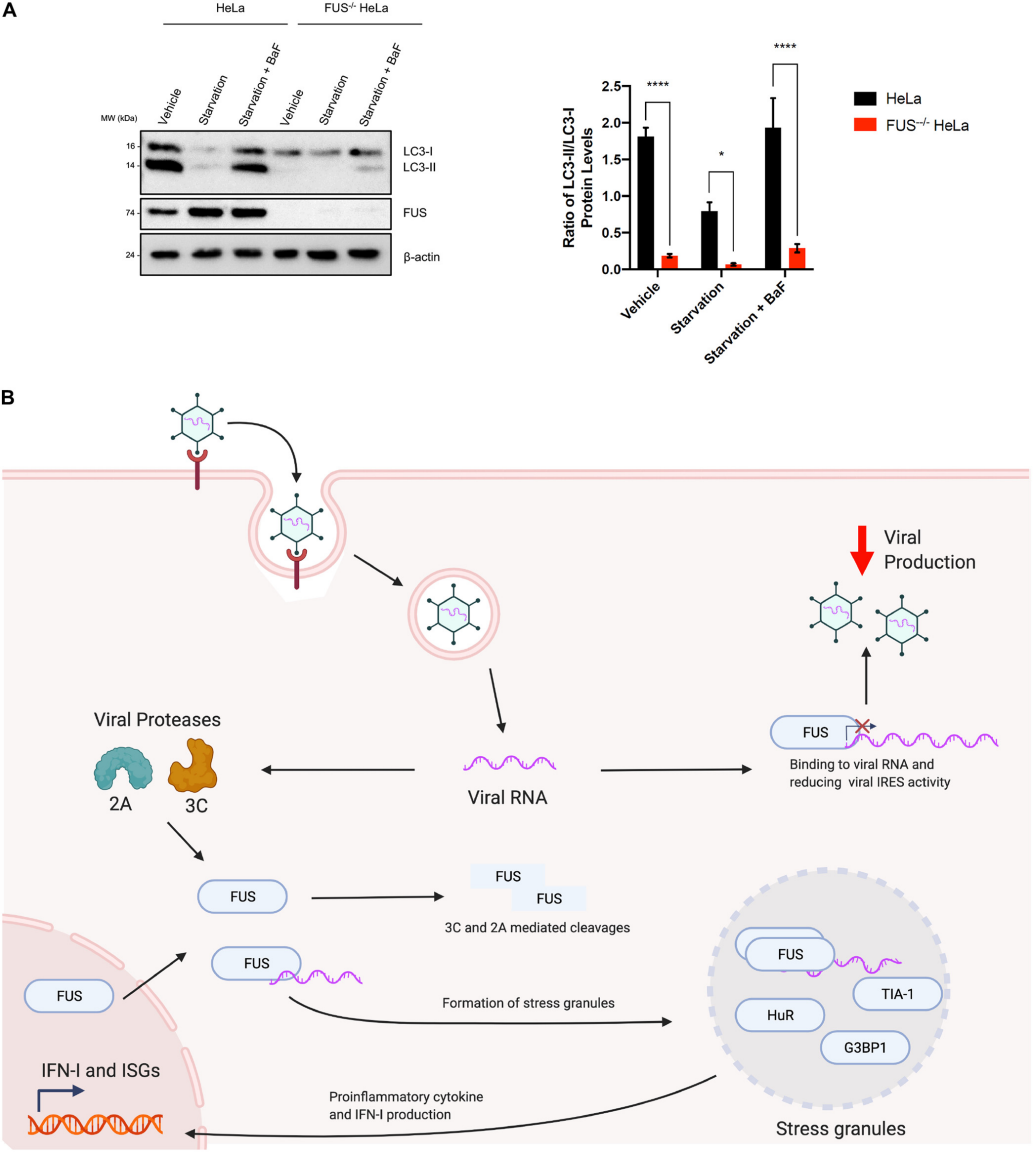
Knockout of FUS inhibits autophagy. (A) Control and FUS^−/−^ HeLa cells were treated with vehicle, starvation medium (HBSS) for 4 h, or starvation medium plus BaF (200 nM BaF in HBSS) for 4 h. Western blot analysis of LC3, FUS, and β-actin was performed. Levels of LC3-I and LC3-II were quantified by densitometric analysis using Fiji image analysis software, and ratios of LC3-II levels to LC3-I levels are presented on the right (*n* = 3). Statistical analysis was performed by unpaired Student's *t* test. *, *P* < 0.05; ****, *P* < 0.0001. (B) Schematic diagram for the proposed antiviral mechanism of FUS. FUS suppresses viral replication through direct interaction with the viral RNA and by inhibition of viral RNA transcription and IRES-mediated translation, while at the same time mediating the formation of SGs and promoting antiviral proinflammatory cytokine and IFN-I production. To counter this suppressive influence, viral proteases (3C and 2A) mislocalize FUS from its nuclear location and further cleave it.

CVB3 is known to hijack the cellular autophagy machinery to promote its replication (26, 27). To address whether FUS inhibits viral growth partly by regulating autophagy, we examined the effects of FUS deletion on autophagy using control and FUS^−/−^ HeLa cells. Cells were either starved with Hanks’ balanced salt solution (HBSS) medium (an inducer of autophagy) or treated with bafilomycin A1 (which inhibits cellular autophagy by inhibiting the acidification of lysosomes and the fusion of autophagosomes and lysosomes). Similar to the findings in a recent report by Arenas et al. (28), we observed that FUS^−/−^ led to suppressed autophagy, as evidenced by a significant decrease in LC3-II/LC3-I under both basal and starvation conditions (Fig. 8A). Given the early findings that autophagy is utilized by CVB3 for successful replication (29), it is unlikely that hijacking the autophagy pathway could contribute to the increased viral titer under FUS^−/−^ conditions.

In summary, we propose that FUS exerts antiviral activity against CVB3 through direct interaction and by promoting the formation of SG and the production of IFN-I and inflammatory cytokines. To evade the function of FUS, CVB3 has evolved to mislocalize and cleave this protein (Fig. 8B).

## DISCUSSION

In this study, we identified FUS as a novel host restriction factor against enteroviral infection. We demonstrated for the first time that FUS plays an important role in regulating host innate immune and inflammatory responses to infection. Our data also revealed that FUS is required for the formation of SGs. To counteract the antiviral effects of FUS, CVB3 has developed strategies to mislocalize and cleave FUS, causing its loss of function.

In neurodegenerative diseases such as ALS, ALS-related FUS mutations, predominantly within the nuclear localization signal (NLS) domain, often lead to its cytoplasmic mislocalization (30–32). In the current study, we observed a similar pathological phenotype of FUS in cells infected with CVB3. However, unlike gene mutation-induced FUS mislocalization, which is caused by dysfunctional NLSs, CVB3-encoded protease 2A seems to drive this abnormal phenomenon through cleavage of key proteins in the nuclear pore complex (4, 14, 15). Upon FUS accumulation in the cytoplasm, we found that both viral proteases 3C and 2A target FUS for degradation to generate 45-kDa and 55-kDa protein fragments, respectively, with the latter being more abundant.

Often, cleavage/degradation of cellular proteins during viral infection hints at a potential antiviral role of these proteins, as this effect may represent a viral strategy to attenuate the negative impact of host restriction factors to achieve successful infection. In the case of FUS, we found that, similar to other RBPs, such as TDP-43 and AUF1/hnRNP D (3–8), FUS also has an antiviral role during CVB3 infection. It was observed that the virus titers are significantly higher (>10-fold) in cells with knockout or knockdown of FUS than those in the control cells. A quantitative PCR (qPCR) experiment revealed significant but moderately higher viral RNA levels in FUS^−/−^ cells (∼2-fold) than in control cells. Further investigation demonstrated that FUS also suppresses CVB3 IRES-driven translation, likely through direct binding to viral RNA genome. These results suggest that the higher viral titers (>10-fold) seen in knockout or knockdown of FUS seems to be partially the consequence of FUS’s involvement in both viral RNA expression and viral translation.

In addition to its direct effect on viral replication, the present study also revealed a role for FUS in mediating innate immune responses under multiple pathological stimulations. We observed that CVB3-, poly(I·C)-, and LPS-induced gene expression of IFN-I and proinflammatory cytokines is largely impaired in FUS^−/−^ cells compared to control cells, suggesting a regulatory role for FUS in IFN-I and proinflammatory cytokine production. Interestingly, a recent study reported that upon IFN-β treatment, FUS mRNA stability is enhanced, leading to an accumulation of FUS protein (33). Our data together with this finding support a close relationship between FUS and the host IFN-I innate immune response. In conjunction with the proposed antiviral role of SGs (23, 24), we found that, among several known SG-inducing stimulants, CVB3 and poly(I·C) were unable to induce SG formation in FUS^−/−^ cells, indicating a role for FUS in regulating SG formation in response to RNA viral infection. The exact mechanism underlying this observation remains unclear. The key kinase responsible for SG formation during the two stimulants is PKR (protein kinase R), which is encoded by an ISG that is expressed during antiviral immune responses and shown to be colocalized within the SGs during viral infection (24). We thus speculate that an impaired PKR pathway, as a result of decreased production of IFN-I in FUS^−/−^ cells, contributes, at least in part, to the observed failure to form SGs.

While our data in the current study suggest that full-length FUS plays an antiviral role, the functions of the two cleavage products (45 kDa and 55 kDa), generated after CVB3 infection, remain unclear. Future investigation is warranted to further understand the potential effects of these cleavage products on mediating antiviral innate immune response and/or viral replication processes. These investigations would require first overexpressing the individual protein products before measuring the effects on antiviral immune responses after poly(I·C) stimulation and on viral titers after CVB3 infection.

In conclusion, the present study offers evidence of the roles of FUS in suppressing viral replication via (i) moderately inhibiting viral RNA expression, (ii) reducing CVB3 IRES-mediated translation, and (iii) mediating antiviral innate immune responses, which occurs partially through SG formation. In an attempt to mitigate this antiviral effect, viral proteases (3C and 2A) target FUS for cleavage and degradation. We believe that the findings from this study also provide new insights into molecular phenotype and potential implication of FUS loss of function in the pathogenesis of neurodegenerative diseases, where cytoplasm-localized FUS has been regarded as one of the pathological phenotypes of disease, and the involvement of inflammation in neurodegeneration has been more widely studied in recent years.

## MATERIALS AND METHODS

### Cell culture and viral infection.

All cells used in this study, including HEK293T and HeLa cells (American Type Culture Collection), were cultured in Dulbecco’s modified Eagle’s medium (DMEM) supplemented with 10% fetal bovine serum (FBS) and 100 μg/ml penicillin/streptomycin. FUS^−/−^ HeLa cells were generously provided by Marc-David Ruepp at King’s College London (London, UK) (34). NSC-34 and SH-SY5Y cells were gifts from Neil Cashman at the University of British Columbia (Vancouver, Canada) (35).

CVB3 (Kandolf strain) infection was performed by incubating the viruses at the indicated multiplicity of infection (MOI) in serum-free DMEM for 1 h; then, the medium was switched back to FBS-containing DMEM, and cells were incubated for the periods of time shown in the figure legend.

### Reagents and treatments.

Stress granule formations were induced in cells as follows: treatment with 0.5 mM arsenite (Sigma-Aldrich; S7400) for 1 h, 10 μM MG132 (Sigma-Aldrich; C2211) for 3 h, starvation in Hanks’ balanced salt solution (HBSS; Invitrogen; 14175095) for 6 h, 4 μM thapsigargin (Sigma-Aldrich; T9033) for 1 h, 1 μg/ml tunicamycin (Sigma-Aldrich; T7765) for 6 h, and infection with CVB3 at an MOI of 10 for 3 h or transfection with 1 μg/ml high-molecular-weight poly(I·C) (InvivoGen; tlrl-pic-5) using Lipofectamine RNAiMAX (Invitrogen; 13778075) for 6 h. Autophagy was induced by incubating cells with HBSS medium for 4 h with or without a 200 nM concentration of the vacuolar H^+^ ATPase inhibitor bafilomycin A1 (BaF) (Sigma-Aldrich; B1793) for 4 h.

### Plasmids and siRNA transfection.

The wild-type CVB3-3C (3C^wt^) and C147A mutant CVB3-3C (3C^mut^) constructs were generous gifts from Carolyn Coyne at the University of Pittsburgh (36). CVB3-2A (2A^wt^) construct was established as described in our previous publication (37). The C49-CVB3-5′UTR dual luciferase plasmid was a gift from Decheng Yang at the University of British Columbia (20). The hemagglutinin (HA)-tagged wild-type (WT) FUS plasmid was provided by Neil Cashman at the University of British Columbia (38). The WT green fluorescent protein (GFP)-FUS plasmid was a gift from Randal Tibbetts at the University of Wisconsin (39). The siRNAs targeting FUS (sc-40563) and the scrambled siRNAs (sc-37007) were purchased from Santa Cruz Biotechnology. For transfection, cells were transiently transfected with plasmid cDNAs with either Lipofectamine 2000 (Invitrogen; 11668-019) or DNAfectin Plus (Abmgood; G2500), while siRNAs were transfected using Lipofectamine RNAiMAX (Invitrogen; 13778075) following the manufacturer’s instructions.

### Western blot analysis.

Cells were lysed in lysis buffer (10 mM HEPES [pH 7.4], 50 mM Na pyrophosphate, 50 mM NaF, 50 mM NaCl, 5 mM EDTA, 5 mM EGTA, 100 μM Na_3_VO_4_, 0.1% Triton X-100, and protease inhibitors), and Western blotting was conducted using the following primary antibodies: FUS (Proteintech no. 11570-1-AP), VP1 (Mediagnost no. M47), TDP-43 (Proteintech no. 10782-2-AP), cleaved caspase 3 (Cell Signaling Technology no. 9661), GAPDH (Cell Signaling Technology no. 14C1), LC3B (Cell Signaling Technology no. 3868), and β-actin (Santa Cruz Biotechnology no. sc-47778).

### *In vitro* and *in vivo* cleavage assays.

The *in vitro* cleavage assay was performed by incubating HeLa cell lysates (30 μg) with different amounts of purified CVB3 proteases 3C or 2A in cleavage assay buffer (20 mM HEPES [pH 7.4], 150 mM potassium acetate, and 1 mM dithiothreitol [DTT]) for indicated times at 37°C. The reaction was stopped with the addition of 6×SDS sample buffer at 95°C for 10 min, followed by Western blot analysis of FUS cleavage (40). The *in vivo* cleavage assay was performed as previously described (4). Briefly, cells were transfected with 3C^wt^ or 3C^mut^ plasmid for 24 h, followed by Western blot analysis of FUS cleavage.

### Virus titration assay.

Cell culture supernatants were serially diluted and then loaded onto 60-well Terasaki plates seeded with HeLa cells. After 48 h of incubation, the 50% tissue culture infective dose titer (TCID_50_) was calculated by the statistical method of Reed and Muench (41). Virus titers were later recalculated as PFU per milliliter by using the formula 1 infectious unit = 0.7 TCID_50_, as described previously (42).

### Luciferase assay.

Cells were transfected with C49-CVB3-5′UTR luciferase plasmid for 24 h, followed by mock infection or CVB3 infection for 5 h. Luciferase activities were measured with the dual-luciferase reporter assay system (Promega; E1910) as per the manufacturer’s instructions. The C49-CVB3-5′UTR reporter plasmid was constructed to contain two tandem open reading frames encoding an upstream SV40-driven *Renilla* luciferase (serving as an internal cap-dependent translation control) and a downstream CVB3-5′ UTR-driven firefly luciferase that monitors cap-independent translation (20).

### RNA extraction and qPCR.

Cellular RNA was extracted using the Monarch total RNA miniprep kit (New England Biolabs; T2010) following the manufacturer’s instructions. mRNA level was measured via quantitative reverse transcription-PCR (qRT-PCR) using the Luna universal one-step qRT-PCR kit (New England Biolabs; E3005) on a ViiA 7 real-time PCR system (Applied Biosystems). The primer pairs used for mRNA measurement are as follows: *CVB3 2A* (forward, 5′-GCT TTG CAG ACA TCC GTG ATC-3′; reverse, 5′-CAA GCT GTG TTC CAC ATA GTC CTT CA-3′), *GAPDH* (forward, 5′-AAT CCC ATC ACC ATC TTC CA-3′; reverse, 5′-TGG ACT CCA CGA CGT ACT CA-3′), *IFNB1* (forward, 5′-CTT GGA TTC CTA CAA AGA AGC AGC-3′; reverse, 5′-TCC TCC TTC TGG AAC TGC TGCA-3′), *TNFA* (forward, 5′-CTC TTC TGC CTG CAC TTT G; reverse, 5′-ATG GGC TAC AGG CTT GTC ACT C-3′), *IL-6* (forward, 5′-AGA CAG CCA CTC ACC TCT TCA G-3′; reverse, 5′-TTC TGC CAG TGC CTC TTT GCT G-3′), *IL1B* (forward, 5′-CCA CAG ACC TTC CAG GAG AAT G-3′; reverse, 5′-GTG CAG TTC AGT GAT CGT ACA GG-3′), *CCL5* (forward, 5′-CCT GCT TTG CCT ACA TTG C-3′; reverse, 5′-ACA CAC TTG GCG GTT CTT TCG G-3′), *CXCL10* (forward, 5′-GGT GAG AAG AGA TGT CTG AAT CC-3′; reverse, 5′-GTC CAT CCT TGG AAG CAC TGC A-3′), *MX1* (forward, 5′-GGC TGT TTA CCA GAC TCC GAC A-3′; reverse, 5′-CAC AAA GCC TGG CAG CTC TCT A-3′), *TNFAIP3* (forward, 5′-CTC AAC TGG TGT CGA GAA GTC C-3′; reverse, 5′-TTC CTT GAG CGT GCT GAA CAG C-3′), and *β-actin* (forward, *5′-*CAC CAT TGG CAA TGA GCG GTT C-3′; reverse, 5′-AGG TCT TTG CGG ATG TCC ACG T-3′).

### Immunofluorescence and confocal microscopy.

Cells on coverslips were first fixed with 5% paraformaldehyde solution and permeabilized with 1% Triton X-100 before being blocked for 1 h with 3% bovine serum albumin. Cells were then incubated with primary antibodies at 4°C overnight and then with the corresponding secondary antibodies for 1 h. The primary antibodies used are those specific for FUS (Proteintech no. 11570-1-AP), G3BP1 (Proteintech no. 13057-2-AP), TIA-1 (Proteintech no. 12133-2-AP), HuR (Proteintech no. 11910-1-AP), HA (Santa Cruz Biotechnology no. sc-7392), VP1 (Mediagnost no. M47), and dsRNA (English and Scientific Consulting kft no. 10010200). Afterward, the coverslips were mounted using Fluoroshield with DAPI (Sigma-Aldrich; F6057). Images were then captured with a Zeiss LSM 880 inverted confocal microscope. Stress granule size was measured using Zeiss Zen v3.1.0 by recording the sizes of 100 granules/condition, with each condition having 3 biological replicates.

### Viral RNA-IP.

After mock or CVB3 (MOI, 10) infection for 5 h, cells in 100-mm culture dishes were lysed in nuclear isolation buffer (1.28 M sucrose, 40 mM Tris [pH 7.5], 20 mM MgCl_2_, and 4% Triton X-100). After centrifugation, the pellets were resuspended in RNA-protein immunoprecipitation (RNA-IP) buffer (150 mM KCl, 25 mM Tris [pH 7.4], 5 mM EDTA, 0.5 mM DTT, 0.5% NP-40, and 100 U/ml RNase inhibitor), and the solution was sheared with a 25-gauge needle. After centrifugation, the supernatant was taken out and subsequently incubated overnight at 4°C with either 5 μg IgG control antibody or FUS antibody (Proteintech no. 11570-1-AP). Afterwards, 50 μl of 50% protein G agarose beads was added and incubated for another 1 h at 4°C before three washes with RNA-IP buffer and one wash with phosphate-buffered saline (PBS). The resulting solution was then subjected to TRIzol (Invitrogen; 15596026) extraction following the manufacturer’s instructions and further analyzed for viral RNA levels using qRT-PCR. The calculation for RNA-IP was performed first by normalizing the RNA levels of immunoprecipitated FUS or IgG control under mock or CVB3 conditions to those of respective input to obtain the Δ*C_T_* for FUS (Δ*C_T_*-FUS) or control (Δ*C_T_*-control). ΔΔ*C_T_* was then calculated by taking the difference of Δ*C_T_*-FUS and Δ*C_T_*-control). The fold enrichment of RNA levels was finally obtained by taking the 2^ΔΔ^*^CT^* for each replicate.

### Statistical analysis.

All Western blot images were quantified by Fiji image analysis software, v1.0. Statistical analysis and the corresponding graphs were done using GraphPad Prism 8 v8.4.0. Details of statistical analysis are provided in the figure legends.
